# A Case Report of High-Risk Percutaneous Coronary Intervention of Left Main Coronary Artery With Cardiogenic Shock

**DOI:** 10.7759/cureus.41983

**Published:** 2023-07-16

**Authors:** Suraj Khanal, Anil K Choudhary, Basant Kumar

**Affiliations:** 1 Cardiology, Post Graduate Institute of Medical Education and Research, Chandigarh, IND; 2 Internal Medicine, Post Graduate Institute of Medical Education and Research, Chandigarh, IND

**Keywords:** intra-aortic balloon pump, percutaneous coronary intervention, cardiogenic shock, acute total occlusion of the left main coronary artery, acute coronary syndrome

## Abstract

Acute total occlusion of the left main artery is a fatal event and is often accompanied by cardiogenic shock. Patients who experience this event have high mortality rates. Early percutaneous coronary intervention (PCI) with hemodynamic support has proven to improve clinical outcomes for these patients. Here we report a case of a 60-year-old man, who came into our emergency room with an acute anterior wall myocardial infarction accompanied by cardiogenic shock. He had a totally occluded left main artery on coronary angiography, necessitating cardiopulmonary resuscitation, followed by PCI with implantation of a drug-eluting stent along with hemodynamic support. Identification of typical ECG changes is crucial in patients with acute coronary syndrome caused by the occlusion of the left main coronary artery. A quick decision to perform a PCI procedure using early circulatory mechanical devices (intra-aortic balloon pump) is critical to patient survival.

## Introduction

Myocardial infarction (MI) owing to the left main coronary artery (LMCA) occlusion constitutes a group of high-risk patients [1]. Acute ST-segment elevation myocardial infarction (STEMI) follows a chain of events commencing with an atherosclerotic plaque rupture, followed by the formation of a thrombus, activation of the coagulation cascade, and platelet aggregation. Acute total occlusion of the left main artery associated with cardiogenic shock (CS) requires rapid complete reperfusion to prevent adverse outcomes [2]. In LMCA patients with stable angina undergoing elective procedures, percutaneous coronary intervention (PCI) has been shown to be non-inferior to coronary artery bypass graft in the absence of disease in other vessels, but for patients with acute MI, revascularization of LMCA remains a difficult procedure to perform [3]. However, for patients who are too seriously ill to tolerate surgery, PCI provides an alternate treatment option [2]. We present the case of a 60-year-old man who arrived in the emergency room with STEMI and CS. The patient had an acute total occlusion of the LMCA that was successfully managed, emphasizing the importance of early PCI with mechanical circulatory support.

## Case presentation

**Figure 1:**
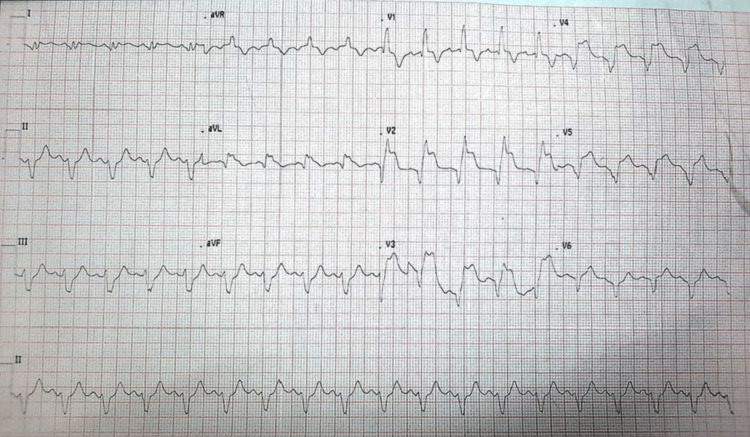
ECG suggesting acute MI of the anterior wall MI, myocardial infarction.

A 60-year-old man with diabetes for the last four years presented with complaints of severe retrosternal chest pain associated with difficulty in breathing and orthopnea for the last 10 hours. His blood sugar at presentation was 140 mg/dl. The patient was in CS with a blood pressure of 72/38 mmHg and the ECG suggested acute MI of the anterior wall (Figure 1).

**Figure 2:**
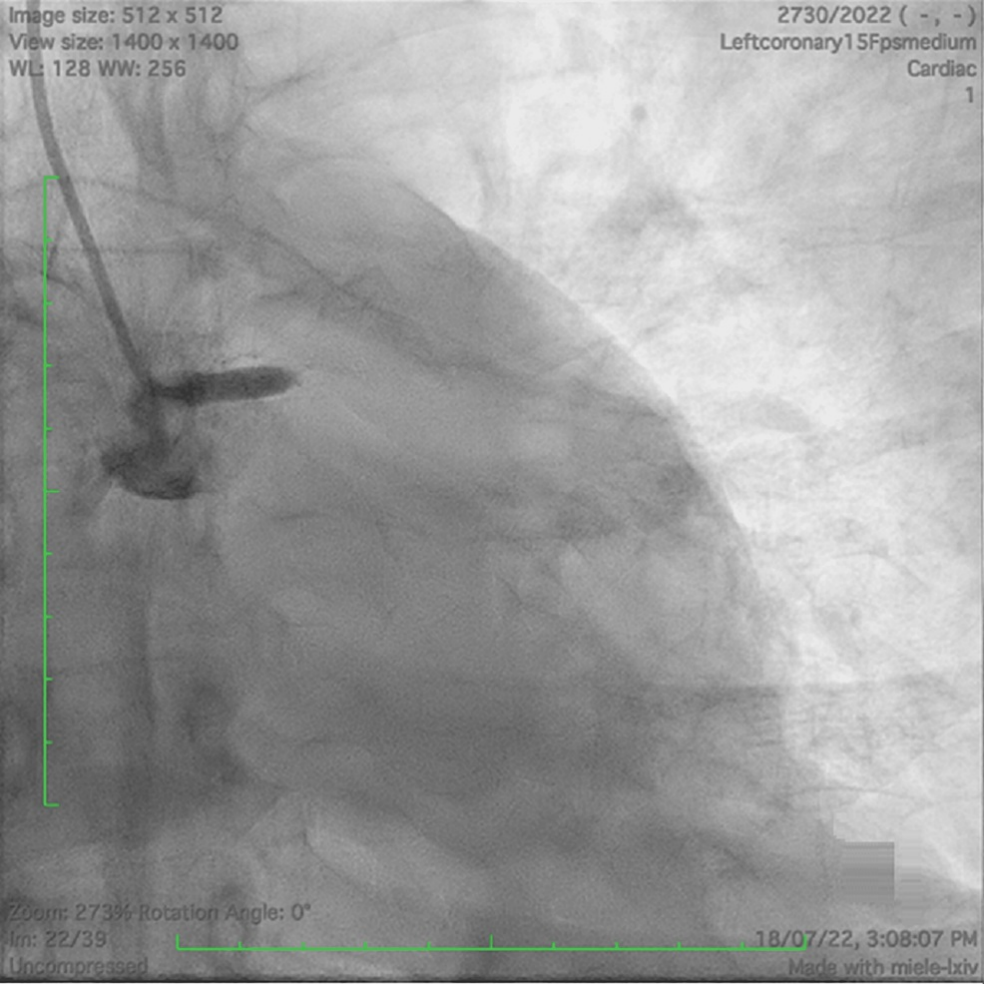
Acute total occlusion of the left main coronary artery

Cardiac troponin T and NT-proBNP (N-terminal pro-brain natriuretic peptide) biomarkers were found to be elevated. The patient was given norepinephrine and oxygen. His oxygen saturation was 94% on 6 l/min of oxygen. Two-dimensional echocardiography showed severe hypokinesia of the territory of the left anterior descending artery (LAD) with an ejection fraction of 20% and no mechanical complications. Coronary angiography revealed total thrombotic occlusion of the LMCA (Figure 2).

**Figure 3:**
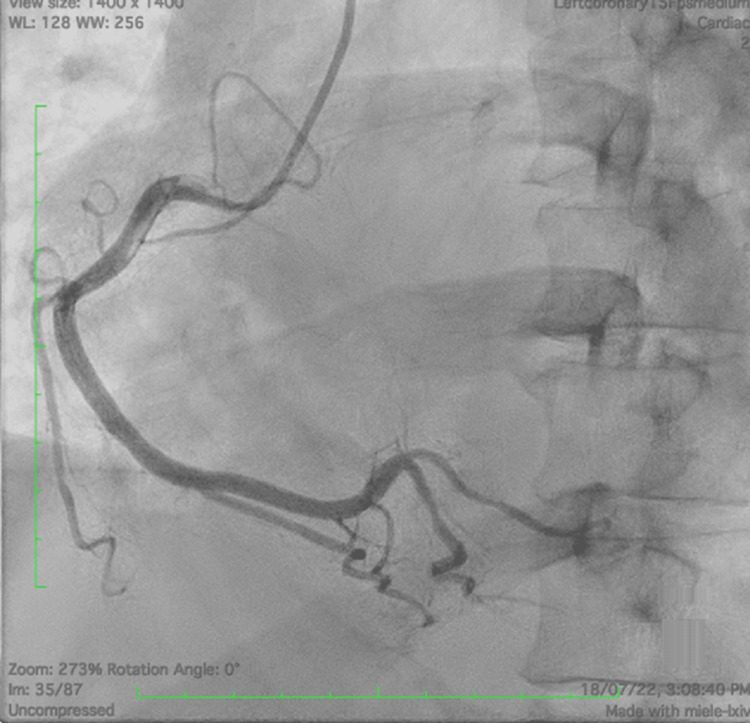
Normal right coronary artery

The patient had a right dominant circulation with a normal right coronary artery providing collateral to the left system, a Rentrop classification of grade 1 (Figure 3).

**Figure 4:**
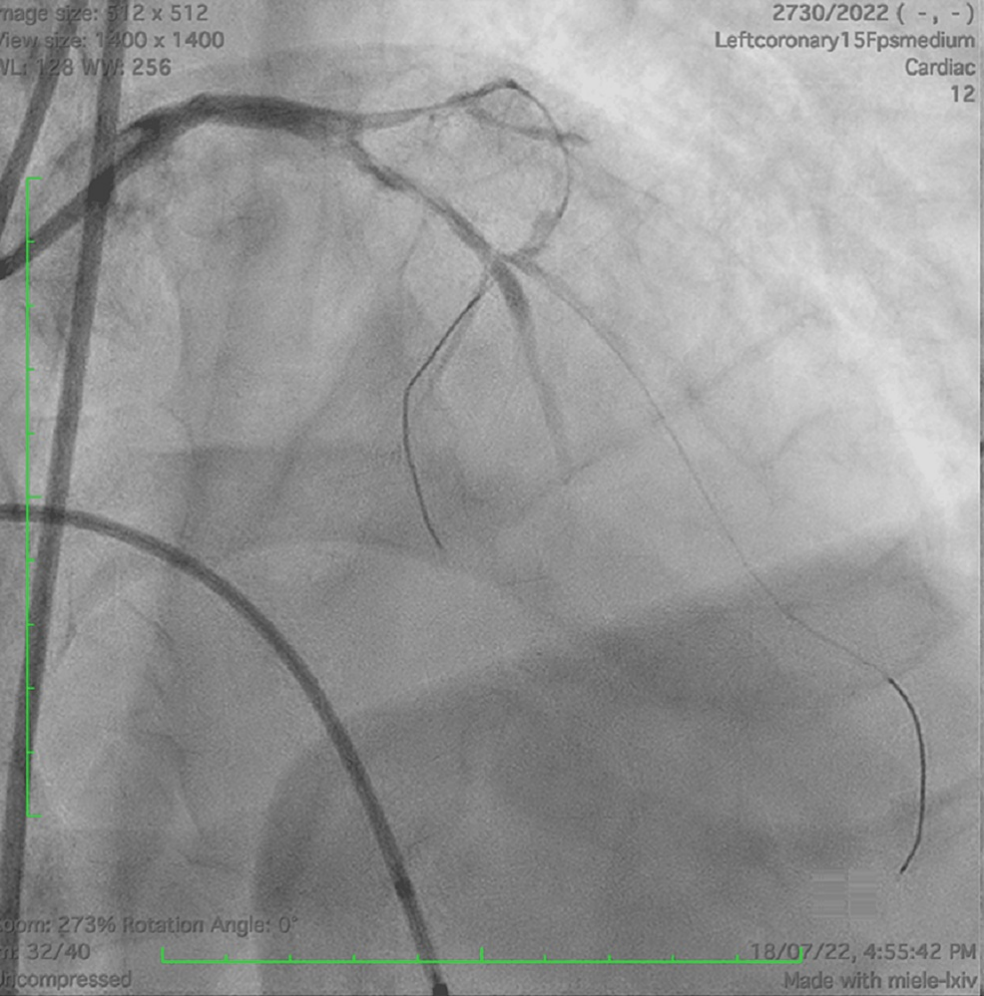
AP cranial view showing wires in both left anterior descending artery and left circumflex artery with TIMI 1 flow AP, anteroposterior; TIMI 1,  thrombolysis in myocardial infarction flow grade 1.

Vasopressor support was increased and an intra-aortic balloon pump (IABP) was inserted to provide hemodynamic support. A 7F Asahi SPB 3.5 guiding catheter was used for percutaneous transluminal coronary angioplasty of LMCA. The LAD and left circumflex artery (LCx) were wired with the Fielder FC wire (Figure 4).

**Figure 5:**
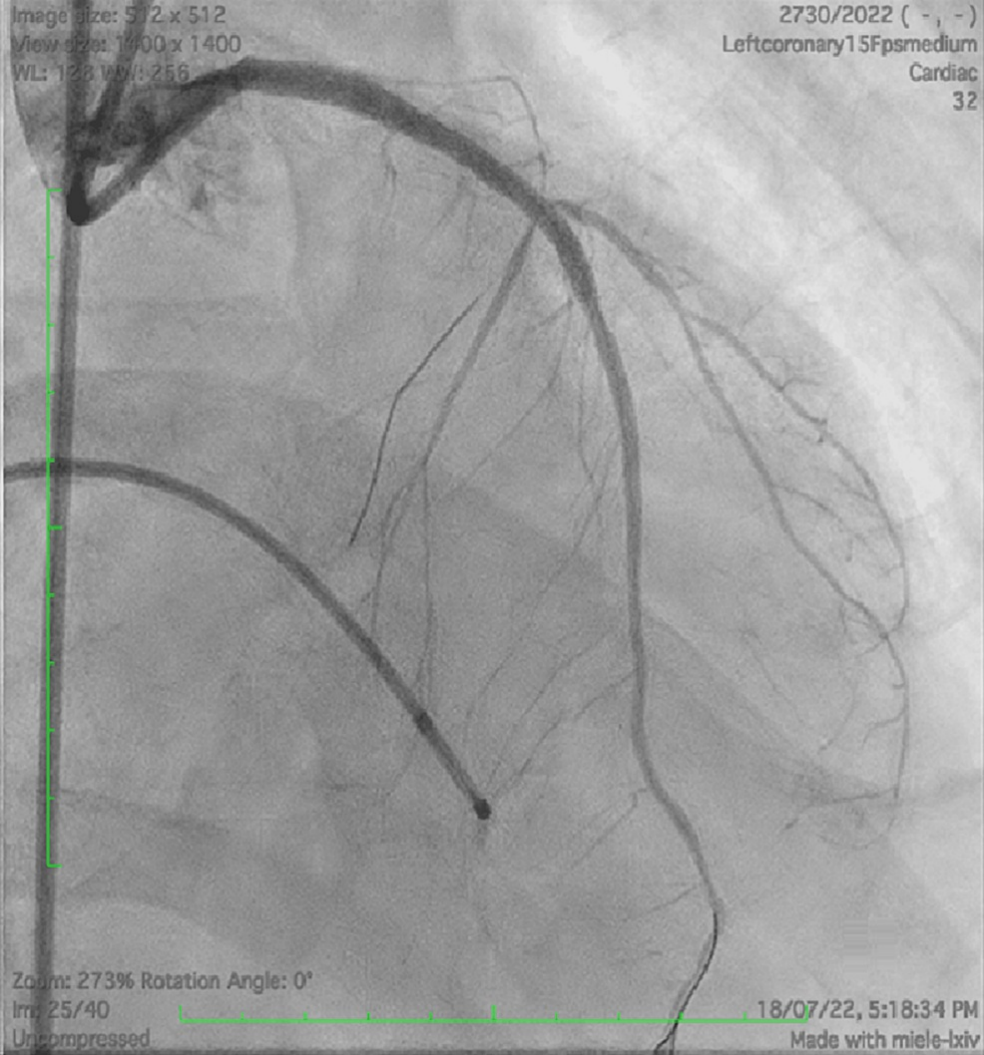
TIMI 3 flow rate after stenting of LM-LAD TIMI 3, thrombolysis in myocardial infarction flow grade 3; LM-LAD, left main-left anterior descending artery.

The patient developed ventricular tachycardia and received 4 DC shocks; however, the patient developed asystole. The patient was intubated, and cardiopulmonary resuscitation (CPR) was carried out followed by a temporary pacemaker implantation (TPI) as a life-saving procedure since the patient had developed bradycardia and asystole during PCI. Sequential pre-dilatation of LMCA and LAD was done with Ryurei 1×5 mm, 2×15 mm, and 2.5×15 mm balloons. However, coronary blood flow could not be achieved (Figure 4). Thrombosuction was performed using an Aspiron suction catheter and the TIMI 1 (thrombolysis in myocardial infarction flow grade 1) flow rate was achieved. A cocktail of tirofiban with adrenaline and nicorandil was administered through the Aspiron catheter. Left main-left anterior descending artery (LM-LAD) was stented with a drug-eluting stent of size 3×48 mm and the post-dilatation was done with a non-compliant (NC) balloon of size 3×12 mm in LAD. The proximal optimization technique was carried out in LMCA using a 4×10 mm NC balloon. The TIMI 3 flow rate was achieved in LM-LAD (Figure 5).

LCx was most probably a chronic total occlusion lesion as it was getting filled by retrograde collaterals from the right coronary artery. Hemodynamics improved after the procedure and the patient was shifted to the coronary care unit on the vasopressors, IABP, TPI, and ventilator support. The temporary pacemaker and IABP were removed after 24 hours, the vasopressors were tapered, and the patient was extubated after 48 hours. The patient was discharged after four days in a stable hemodynamic condition. The medications prescribed at the time of discharge were aspirin 150 mg, clopidogrel 75 mg, rosuvastatin 20 mg, rabeprazole 20 mg, and spironolactone 25 mg daily. At three months follow-up, the patient was doing well, and the left ventricular ejection fraction improved to 30%.

## Discussion

LMCA disease which is usually associated with multi-vessel coronary artery disease (CAD) is present in about 5-6% of coronary angiography patients [4]. In patients undergoing cardiac catheterization, the incidence of STEMI caused by LMCA ranges between 0.8% and 2.5% [2,5,6]. Since it feeds much of the myocardium, the acute occlusion of LMCA usually presents as a massive infarction [7]. It causes major complications like CS, arrhythmias, and rapid hemodynamic deterioration, which result in adverse outcomes. Our patient presented unfavorably as CS, ventricular arrhythmia, and asystole, requiring CPR and mechanical ventilation. Total acute occlusion of LMCA is considered a high-risk angiographic observation and has a higher mortality rate than other lesions. This was highlighted in a meta-analysis of 13 studies, in which 26% of STEMI patients with LMCA had CS and a significantly higher 30-day all-cause mortality [8]. To date, insufficient evidence exists to describe strategies for managing acute total occlusion of LMCA. The PCI for stable LMCA has proven to be an effective therapeutic intervention in recent years [9]. Percutaneous revascularization with circulatory support, primarily in the form of an IABP or Impella, has proven to be a more conducive strategy than surgical options [10]. With an in-hospital mortality rate of 31%-58% and a 30-day mortality rate of 36%-63%, there is still a poor prognosis for STEMI caused by LMCA disease [2,5,6,11,12]. A quick attempt to open the left main stem as quickly as possible is the key to restoring flow to the left coronary branches. It has been pointed out that for STEMI treated with primary angioplasty, right coronary dominance provides a better prognosis than left dominance as a result of a significant amount of vasculature feeding to the left ventricle [13]. In addition, TIMI flow grades have an important prognostic impact on patients undergoing primary angioplasty in LMCA [14]. Although a TIMI flow grade of 3 is difficult to achieve in this scenario, every effort must be made to achieve it, as was done in our case. Our patient had a right dominant circulation as shown in angiographic images and we achieved TIMI 3 flow, thus leading to a favorable outcome. The need for circulatory support is necessary for close to 90% of patients presenting with manifest CS [9]. Therefore, circulatory support in the form of IABP or Impella plays a major role in managing this condition as seen in our patient. Our patient received adequate intensive care following the procedure, which was crucial to achieving positive outcomes.

## Conclusions

Although uncommon, LMCA total occlusion in acute coronary syndrome is a rare yet high-risk angiographic finding. Despite effective PCI-assisted recanalization, the mortality rate is still high in this subgroup of patients. Early recognition of typical ECG changes in patients with ACS caused by LMCA occlusion and quick decision to perform a PCI procedure using circulatory mechanical devices such as of IABP or Impella is crucial for patient survival.
